# Primed Avian Mesenchymal Stem Cell-Derived Small Extracellular Vesicles Restore Granulosa Cell Homeostasis in a CTX-Induced POI-like Dysfunction Model Under Human Menopausal Gonadotropin Stimulation

**DOI:** 10.3390/ijms27114934

**Published:** 2026-05-29

**Authors:** Hsiang-Chun Dong, Kuo-Min Su, Chi-Kang Lin, Hui-Rong Cheng, Chih-Hsiang Yin, Fung-Wei Chang

**Affiliations:** 1 Department of Obstetrics and Gynecology, Tri-Service General Hospital and National Defense Medical University, Taipei 11490, Taiwan; surgjimmy@yahoo.com.tw (H.-C.D.); aeolusfield@hotmail.com (K.-M.S.); kung568@gmail.com (C.-K.L.); candyjung@mail2000.com.tw (H.-R.C.); j19900302@gmail.com (C.-H.Y.); 2 Graduate Institute of Medical Sciences, National Defense Medical University, Taipei 11490, Taiwan; 3 Department of Obstetrics and Gynecology, School of Medicine, College of Medicine, National Defense Medical University, Taipei 11490, Taiwan; 4 Department of Periodontology, School of Dentistry, Tri-Service General Hospital, National Defense Medical University, Taipei 11490, Taiwan; 5 Division of Obstetrics and Gynecology, Tri-Service General Hospital Penghu Branch, National Defense Medical University, Penghu 880026, Taiwan; 6 Department of Obstetrics and Gynecology, Taipei City Hospital, Taipei 103212, Taiwan

**Keywords:** granulosa cells, premature ovarian insufficiency, cyclophosphamide model, primed small extracellular vesicles, avian mesenchymal stem cells, anti-Müllerian hormone, human menopausal gonadotropin, mitochondrial membrane potential

## Abstract

Premature ovarian insufficiency (POI) is a heterogeneous disorder, and cyclophosphamide (CTX)-induced ovarian injury represents an acquired POI-like condition rather than the full clinical spectrum of POI. In this study, CTX was used to establish a granulosa cell dysfunction model to evaluate whether primed avian mesenchymal stem cell-derived small extracellular vesicles (primed AMSC-sEVs) could restore granulosa cell homeostasis under human menopausal gonadotropin (hMG) stimulation, a clinically relevant gonadotropin context used in ovarian stimulation. Human granulosa cells were exposed to CTX and subsequently treated with hMG, naïve AMSC-sEVs, or primed AMSC-sEVs. Cell viability, mitochondrial membrane potential, *AMH* and *FSHR* expression, and hormone secretion were examined. CTX reduced cell viability, mitochondrial membrane potential, *AMH* and *FSHR* expression, and AMH/estradiol secretion, confirming the establishment of a POI-like granulosa cell dysfunction state. hMG partially restored selected functional markers but showed limited effects on cell viability and mitochondrial recovery. In contrast, primed AMSC-sEVs markedly restored granulosa cell viability, mitochondrial membrane potential, AMH and FSHR expression, and endocrine output, with stronger effects than naïve AMSC-sEVs. Co-treatment with hMG did not consistently enhance the restorative effects of primed AMSC-sEVs beyond those achieved by primed AMSC-sEVs alone. These findings indicate that primed AMSC-sEVs primarily act by restoring granulosa cell and mitochondrial homeostasis, thereby supporting the cellular competence required for gonadotropin responsiveness in an acquired POI-like condition.

## 1. Introduction

Premature ovarian insufficiency (POI) is a disorder characterized by loss of ovarian activity before the age of 40 years and is clinically associated with menstrual disturbance, elevated gonadotropins, and reduced estradiol levels [1]. Its clinical implications extend well beyond infertility, encompassing vasomotor symptoms, impaired sexual health, reduced bone density, adverse cardiometabolic outcomes, and substantial psychosocial burden. Contemporary clinical guidance therefore frames POI as a chronic reproductive and endocrine disorder rather than a condition defined solely by failure to conceive [2].

At the ovarian level, POI reflects progressive impairment of follicular integrity and function. Among the cellular compartments involved, granulosa cells are central to maintenance of the follicular microenvironment, coordination of endocrine signaling, support of oocyte competence, and preservation of gonadotropin responsiveness [2]. Functional deterioration of granulosa cells is therefore not a secondary event, but a key pathological component of ovarian insufficiency. This concept is particularly relevant for experimental studies, because granulosa cell-based in vitro models allow direct assessment of ovarian functional decline and early evaluation of candidate restorative strategies under controlled conditions [1,3].

Anti-Müllerian hormone (AMH) is one of the most informative functional indicators in this setting. AMH is produced by granulosa cells of small growing follicles [4,5], and serum AMH levels correlate strongly with the number of developing follicles, which is why AMH is widely used as a marker of ovarian reserve [6,7]. In cell-based models, reduction in AMH expression can therefore be interpreted as evidence of compromised granulosa cell competence and disturbed follicular support rather than as an isolated endocrine fluctuation [7]. For studies aimed at validating recovery of ovarian function in a POI-like state, AMH is consequently a biologically appropriate primary endpoint [8].

The role of granulosa cell responsiveness to gonadotropic stimulation is likewise relevant. Impairment of follicle-stimulating hormone receptor (FSHR) signaling is biologically meaningful because functional defects in FSHR have been directly linked to hypergonadotropic ovarian failure in humans [9]. In the context of ovarian insufficiency, decreased FSHR expression or responsiveness may therefore reflect loss of granulosa cell functional identity and reduced capacity to respond to hormonal cues [9,10,11]. Assessment of AMH together with FSHR-related responsiveness is accordingly well suited to characterize the functional state of injured granulosa cells and to determine whether a given intervention restores endocrine competence rather than merely preserving cell survival.

Cyclophosphamide (CTX) is widely used in experimental ovarian research because it reproducibly induces ovarian injury with features relevant to POI. Although CTX is clinically recognized for its gonadotoxicity, its value in mechanistic studies lies in its ability to provoke granulosa cell dysfunction, suppress ovarian functional markers, and disrupt intracellular homeostasis under controlled experimental conditions [1,12,13]. Experimental evidence indicates that CTX exposure promotes oxidative injury, perturbs reactive oxygen species homeostasis, and impairs mitochondrial function in ovarian granulosa cells [13]. In vivo and in vitro studies have further shown that CTX-induced ovarian damage is accompanied by mitochondrial oxidative stress, pyroptotic or ferroptotic injury, and deterioration of follicular support [14]. These properties make CTX a practical inducer for establishing a POI-like granulosa cell dysfunction model when the objective is to evaluate restoration of ovarian cellular function under sustained stress [14,15,16]. Nevertheless, CTX-induced injury represents an acquired and experimentally tractable form of ovarian insufficiency and does not recapitulate the full etiologic spectrum of clinical POI, particularly genetically determined forms.

Oxidative stress and mitochondrial dysfunction are increasingly recognized as major mechanistic drivers of ovarian decline [3,13,17]. Reviews in this field have emphasized that oxidative imbalance accelerates ovarian aging and insufficiency through promotion of apoptosis, inflammation, mitochondrial damage, telomere attrition, and broad biomolecular injury [3]. In granulosa cells, mitochondrial function is critical because these cells require adequate ATP production, redox balance, and mitochondrial membrane potential to sustain endocrine responsiveness and follicular support. Loss of mitochondrial membrane potential reflects mitochondrial depolarization and is closely associated with impaired cellular homeostasis, reduced viability, and compromised hormone-producing capacity. Accordingly, restoration of mitochondrial membrane potential was considered a mechanistically relevant endpoint in the present study, linking mitochondrial recovery to AMH/FSHR-related granulosa cell competence and endocrine function in the CTX-induced POI-like model [17].

Human menopausal gonadotropin (hMG), which contains both FSH and LH activity, is commonly used to stimulate ovarian cells [18]. In the present study, hMG was used to represent a clinically relevant gonadotropin stimulation context rather than as the sole therapeutic driver. This design reflects the clinical situation in which gonadotropins are used to promote follicular development, but their effectiveness depends on the residual functional state of granulosa cells. Under this framework, restoration of AMH and related functional markers in the presence of hMG would indicate that the injured granulosa cells remain biologically responsive and that an adjunctive intervention may improve this residual competence. This rationale is especially pertinent when the experimental goal is to verify alleviation of POI-like dysfunction rather than to model ovulation induction or steroidogenesis in isolation [2,9].

Small extracellular vesicles (sEVs) are nanosized membrane-bound vesicles released by cells and function as mediators of intercellular communication through the transfer of proteins, lipids, and regulatory RNAs to recipient cells. sEVs have emerged as a promising cell-free platform for tissue repair and intercellular regulation [19,20]. By delivering proteins, lipids, mRNAs, and regulatory microRNAs, stem cell-derived extracellular vesicles can influence apoptosis, oxidative stress, inflammation, and mitochondrial homeostasis in recipient cells [21]. In the context of ovarian insufficiency, reviews and meta-analyses of preclinical studies indicate that mesenchymal stem cell-derived extracellular vesicles (MSC-EVs) may improve follicle preservation, attenuate granulosa cell injury, and partially restore hormonal profiles in POI models [22,23]. In the present study, avian mesenchymal stem cells (AMSCs), a specific MSC source, were used to generate naïve and primed AMSC-derived sEVs for functional evaluation in the CTX-induced POI-like granulosa cell model. In addition, original studies in chemotherapy-related ovarian injury have shown that MSC-derived EVs can preserve ovarian structure and function through modulation of apoptosis-related and PI3K/AKT/mTOR-associated pathways [24]. Together, these findings provide a substantive basis for evaluating sEV-based strategies in POI-related ovarian dysfunction.

Building upon our previously published study, which demonstrated that primed AMSC-sEVs more effectively restored granulosa cell proliferation, ovarian function-associated gene expression, steroidogenic activity, and anti-apoptotic signaling than naïve vesicles in a CTX-induced POI-like model, the present work was designed as a focused extension of that therapeutic framework. In the prior study, primed AMSC-sEVs significantly improved AMH, FSHR, and LHCGR expression, restored estradiol and progesterone secretion, and attenuated CTX-induced apoptosis in hGCs, thereby establishing their superior restorative potential under POI-like stress conditions [25]. Primed AMSC-sEVs may therefore be considered as a candidate adjunctive approach that biologically conditions injured granulosa cells, improving mitochondrial integrity and AMH/FSHR-related competence before or during gonadotropin-based stimulation.

The next unresolved issue is whether the restorative advantage of primed AMSC-sEVs can be translated into improved granulosa cell recovery under gonadotropic stimulation, particularly in the presence of hMG, and whether such recovery is accompanied by restoration of mitochondrial integrity under continued CTX exposure. This question is relevant because hMG represents a clinically used gonadotropin stimulus in ovarian stimulation and ovulation induction, yet its effectiveness depends on the residual functional competence of granulosa cells. Recovery of AMH-related granulosa cell function, preservation of FSHR-associated hormonal responsiveness, and normalization of mitochondrial membrane potential therefore provide a more stringent framework for evaluating therapeutic efficacy in POI-like granulosa cell dysfunction. Accordingly, this study used a CTX-induced POI-like granulosa cell dysfunction model to evaluate whether primed avian AMSC-sEVs restore granulosa cell homeostasis under hMG stimulation. The main endpoints included cell viability, mitochondrial membrane potential, AMH and FSHR expression, and AMH/E2 secretion, with the aim of determining whether primed AMSC-sEVs improve the cellular competence required for gonadotropin responsiveness.

## 2. Results

### 2.1. Primed AMSC-sEVs Restores Cell Viability and Mitochondrial Function in CTX-Induced Granulosa Cell Injury

**Figure 1:**
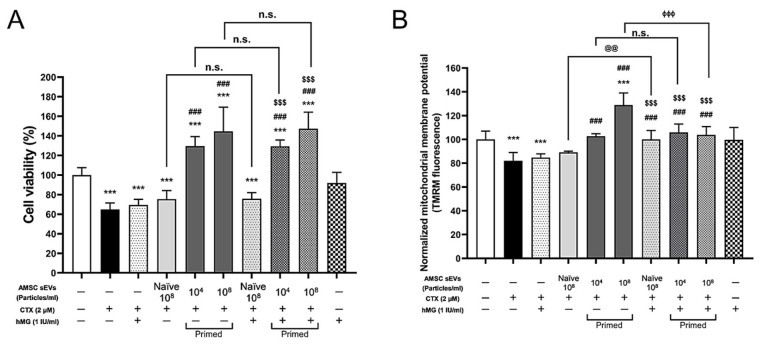
Effects of naïve and primed AMSC-sEVs on cell viability and mitochondrial membrane potential in CTX-treated human granulosa cells. hGCs were treated with cyclophosphamide (CTX, 2 μM) in medium containing 0.25% FBS in the presence or absence of naïve or primed AMSC-derived small extracellular vesicles (sEVs; 10^4^ or 10^8^ particles/mL) and/or human menopausal gonadotropin (hMG, 1 IU/mL). (**A**) Cell viability was measured and expressed as a percentage relative to the untreated control group. (**B**) Mitochondrial membrane potential was quantified using a 96-well plate-based TMRM fluorescence assay and normalized to the untreated control group. Data are presented as mean ± SD (*n* = 6). Statistical significance was determined as follows: *** *p* < 0.001 compared with the control group; ^###^ *p* < 0.001 compared with the CTX-treated group; ^$$$^ *p* < 0.001 compared with the CTX + hMG group; ^@@^ *p* < 0.01 compared with the naïve + CTX group; ^ΦΦΦ^ *p* < 0.001 compared with primed sEVs (10^8^) + CTX group; n.s., not significant. Different bar patterns are used to distinguish the different experimental treatment groups.

Granulosa cell (GC) viability and mitochondrial function were assessed to evaluate the protective effects of primed AMSC-sEVs following CTX-induced injury (Figure 1). Exposure to CTX (2 μM) significantly reduced cell viability to approximately 60–70% of control levels (*p* < 0.001) and decreased mitochondrial membrane potential (MMP) to approximately 80–85% of control (*p* < 0.001), confirming effective induction of cellular and mitochondrial impairment. Treatment with human menopausal gonadotropin (hMG, 1 IU/mL) alone did not significantly improve either cell viability or MMP compared with the CTX group. Likewise, naïve AMSC-sEVs (10^8^ particles/mL) showed limited effects under CTX-treated conditions.

In contrast, primed AMSC-sEVs markedly improved GC viability and MMP under CTX exposure. Primed AMSC-sEVs at 10^4^ particles/mL significantly increased cell viability to approximately 125–135% of control (*p* < 0.001 vs. CTX), while treatment at 10^8^ particles/mL further increased viability to approximately 140–150% (*p* < 0.001 vs. CTX). A similar pattern was observed for mitochondrial function, with primed AMSC-sEVs restoring MMP to near control levels at 10^4^ particles/mL and increasing MMP to approximately 125–130% at 10^8^ particles/mL (*p* < 0.001 vs. CTX).

Co-treatment with hMG did not provide an additional benefit over primed AMSC-sEV treatment alone. Although the combined treatment remained significantly higher than the CTX + hMG group, no significant differences were observed between primed AMSC-sEV-treated groups in the presence or absence of hMG. In the MMP assay, the 10^8^ particles/mL primed AMSC-sEV group showed a lower value when combined with hMG than when administered alone, further indicating that hMG did not enhance the protective effect of primed AMSC-sEVs under these conditions.

Collectively, these results indicate that primed AMSC-sEVs effectively attenuate CTX-induced GC injury by restoring cell viability and mitochondrial membrane potential, whereas co-treatment with hMG does not confer additional rescue beyond that achieved by primed AMSC-sEVs alone.

### 2.2. Primed AMSC-sEVs Increase AMH and FSHR Expression in Granulosa Cells

**Figure 2:**
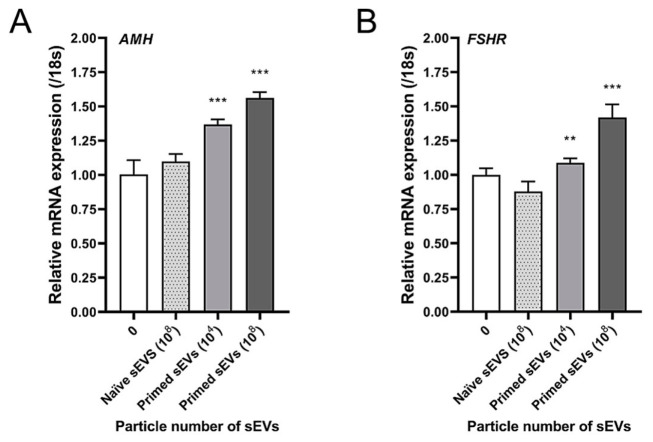
Effects of naïve and primed AMSC- sEVs on AMH and FSHR mRNA expression in human granulosa cells. Human granulosa cells (hGCs) were treated with naïve or primed AMSC-derived small extracellular vesicles (sEVs; 10^4^ and 10^8^ particles/mL). (**A**) Relative mRNA expression of AMH. (**B**) Relative mRNA expression of FSHR. Gene expression levels were quantified by quantitative real-time PCR and normalized to 18S rRNA. Relative expression was calculated using the 2^−ΔΔCt^ method with the untreated control group set as 1. Data are presented as mean ± SD (*n* = 6). (** *p* < 0.01, *** *p* < 0.001 vs. control).

To evaluate the effects of AMSC-derived small extracellular vesicles (sEVs) on GC functional gene expression, the mRNA levels of anti-Müllerian hormone (AMH) and follicle-stimulating hormone receptor (FSHR) were quantified by quantitative real-time PCR following treatment with naïve or primed AMSC-sEVs at different particle concentrations (Figure 2).

Compared with the control group, treatment with naïve AMSC-sEVs (10^8^ particles/mL) did not significantly alter AMH or FSHR expression, indicating limited effects of naïve vesicles under basal conditions. In contrast, primed AMSC-sEVs significantly increased the expression of both AMH and FSHR. Specifically, primed AMSC-sEVs at 10^8^ particles/mL significantly elevated AMH mRNA expression to approximately 1.3–1.4-fold relative to control (*p* < 0.001), while treatment at 10^8^ particles/mL further increased AMH expression to approximately 1.5–1.6-fold (*p* < 0.001). A similar pattern was observed for FSHR expression, where primed AMSC-sEVs at 10^4^ particles/mL significantly increased expression (approximately 1.1-fold, *p* < 0.01), and 10^8^ particles/mL resulted in a greater increase (approximately 1.4–1.5-fold, *p* < 0.001) compared with control.

At equivalent particle concentrations, primed AMSC-sEVs induced higher expression levels of both AMH and FSHR than naïve AMSC-sEVs, supporting enhanced activity following priming.

Collectively, these results show that primed AMSC-sEVs increase the expression of key granulosa cell functional markers, including AMH and FSHR.

### 2.3. CTX Suppresses, While hMG Partially Restores, AMH and FSHR Expression in Granulosa Cells

**Figure 3:**
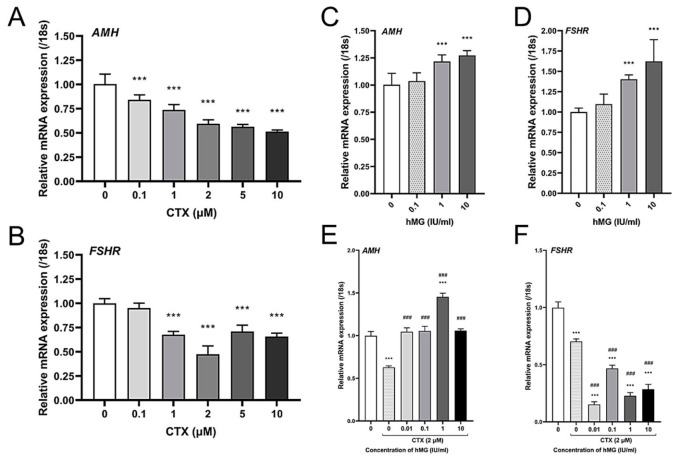
Effects of CTX and hMG on AMH and FSHR mRNA expression in human granulosa cells. hGCs were treated with different concentrations of cyclophosphamide (CTX; 0–10 μM) or human menopausal gonadotropin (hMG; 0–10 IU/mL), either alone or in combination. (**A**,**B**) Relative mRNA expression of AMH and FSHR in hGCs treated with increasing concentrations of CTX (0, 0.1, 1, 2, 5, and 10 μM). (**C**,**D**) Relative mRNA expression of AMH and FSHR in hGCs treated with increasing concentrations of hMG (0, 0.1, 1, and 10 IU/mL). (**E**,**F**) Relative mRNA expression of AMH and FSHR in hGCs co-treated with CTX (2 μM) and increasing concentrations of hMG (0–10 IU/mL). Gene expression levels were quantified by quantitative real-time PCR and normalized to 18S rRNA. Relative expression was calculated using the 2^−ΔΔCt^ method with the untreated control group set as 1. Data are presented as mean ± SD (*n* = 6). (*** *p* < 0.001 compared with the control group; ^###^ *p* < 0.001 compared with the CTX-treated group).

To characterize the effects of cytotoxic stress and gonadotropin stimulation on GC functional gene expression, the mRNA levels of AMH and FSHR were examined following treatment with increasing concentrations of CTX and hMG (Figure 3).

Exposure to CTX resulted in a concentration-dependent reduction in both AMH and FSHR expression. AMH mRNA levels progressively decreased with increasing CTX concentrations (0.1–10 μM), reaching approximately 50–60% of control levels at higher doses (*p* < 0.001) (Figure 3A). A similar trend was observed for FSHR expression, with significant downregulation observed at CTX concentrations ≥ 1 μM (*p* < 0.001) (Figure 3B), confirming effective impairment of GC functional gene expression under cytotoxic conditions.

In contrast, treatment with hMG alone led to an increase in both AMH and FSHR expression. hMG at higher concentrations (1 and 10 IU/mL) significantly elevated AMH mRNA levels compared with control (*p* < 0.001) (Figure 3C). Likewise, FSHR expression was increased in a concentration-dependent manner, with significant upregulation observed at 1 and 10 IU/mL (*p* < 0.001) (Figure 3D).

Under CTX-treated conditions (2 μM), co-treatment with hMG partially restored GC functional gene expression. AMH expression was significantly increased by hMG at multiple concentrations compared with CTX alone (*p* < 0.001), with the greatest increase observed at 1 IU/mL (Figure 3E). Similarly, FSHR expression was elevated by hMG co-treatment relative to CTX alone (*p* < 0.001), although expression levels remained below those of the untreated control group (Figure 3F).

Collectively, these results demonstrate that CTX suppresses AMH and FSHR expression in granulosa cells in a dose-dependent manner, whereas hMG promotes their expression under basal conditions and partially restores their levels under CTX-induced stress.

### 2.4. Primed AMSC-sEVs Restore AMH and FSHR Expression Under CTX-Induced Conditions

**Figure 4:**
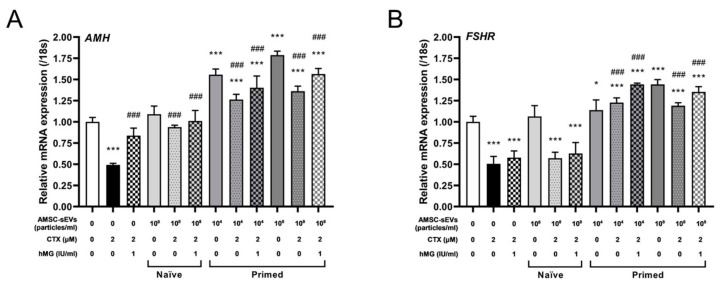
Effects of naïve and primed AMSC-sEVs, alone or in combination with hMG, on AMH and FSHR mRNA expression in CTX-treated human granulosa cells. hGCs were treated with cyclophosphamide (CTX, 2 μM) in medium containing 0.25% FBS, with or without naïve or primed AMSC-derived small extracellular vesicles (sEVs; 10^4^ and 10^8^ particles/mL) and/or human menopausal gonadotropin (hMG, 1 IU/mL). (**A**) Relative mRNA expression of AMH. (**B**) Relative mRNA expression of FSHR. Gene expression levels were quantified by quantitative real-time PCR and normalized to 18S rRNA. Relative expression was calculated using the 2^−ΔΔCt^ method with the untreated control group set as 1. Data are presented as mean ± SD (*n* = 6). (* *p* < 0.05, *** *p* < 0.001 compared with the control group; ^###^ *p* < 0.001 compared with the CTX-treated group). Different bar patterns are used to distinguish the different experimental treatment groups.

To further evaluate the effects of AMSC-derived sEVs under cytotoxic conditions, the expression of AMH and FSHR was assessed in GCs treated with CTX (2 μM) in the presence or absence of naïve or primed AMSC-sEVs and hMG (Figure 4).

Consistent with previous findings, CTX treatment significantly reduced both AMH and FSHR expression compared with the control group (*p* < 0.001). Co-treatment with hMG partially increased AMH expression relative to CTX alone; however, the extent of recovery remained limited. In contrast, naïve AMSC-sEVs (10^8^ particles/mL) produced modest increases in AMH and FSHR expression under CTX-treated conditions, with partial but incomplete restoration.

Notably, primed AMSC-sEVs markedly increased the expression of both AMH and FSHR in CTX-treated GCs. Primed AMSC-sEVs at 10^4^ particles/mL significantly elevated AMH expression compared with the CTX group (*p* < 0.001), and treatment at 10^8^ particles/mL further increased AMH levels to approximately 1.6–1.8-fold relative to control (*p* < 0.001). A similar pattern was observed for FSHR expression, with primed AMSC-sEVs significantly increasing expression at both 10^4^ and 10^8^ particles/mL (*p* < 0.001 vs. CTX), exceeding the levels achieved by naïve AMSC-sEVs at the same concentration.

Co-treatment with primed AMSC-sEVs and hMG also resulted in increased AMH and FSHR expression compared with CTX alone (*p* < 0.001). However, the magnitude of increase was comparable to that observed with primed AMSC-sEVs alone, indicating that hMG did not further enhance the effects of primed AMSC-sEVs under these conditions.

Collectively, these results indicate that primed AMSC-sEVs effectively restore AMH and FSHR expression in CTX-treated granulosa cells, whereas naïve AMSC-sEVs and hMG alone exert relatively limited effects.

### 2.5. Primed AMSC-sEVs Restore AMH and Estradiol Secretion in CTX-Treated Granulosa Cells

**Figure 5:**
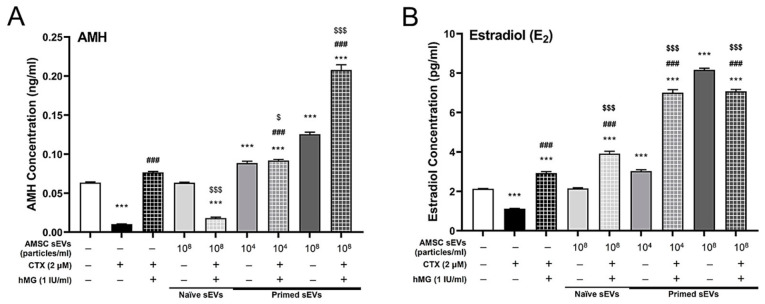
Effects of naïve and primed AMSC-sEVs, alone or in combination with hMG, on AMH and estradiol (E_2_) secretion in CTX-treated human granulosa cells. hGCs were treated with cyclophosphamide (CTX, 2 μM) in medium containing 0.25% FBS, with or without naïve or primed AMSC-derived small extracellular vesicles (sEVs; 10^4^ and 10^8^ particles/mL) and/or human menopausal gonadotropin (hMG, 1 IU/mL). (**A**) Concentration of AMH in culture supernatants. (**B**) Concentration of E_2_ in culture supernatants. Hormone levels were quantified using ELISA according to the manufacturer’s instructions. Data are presented as mean ± SD (*n* = 6). (*** *p* < 0.001 compared with the control group; ^###^ *p* < 0.001 compared with the CTX-treated group; ^$^ *p* < 0.05, ^$$$^ *p* < 0.001 compared with the CTX + hMG group). Actual AMH and E_2_ concentrations are presented as mean ± SD in Supplementary Table S1. Different bar patterns are used to distinguish the different experimental treatment groups.

To further assess the functional recovery of GCs following CTX-induced injury, the secretion levels of AMH and estradiol (E_2_) were quantified in culture supernatants under different treatment conditions (Figure 5).

CTX treatment (2 μM) markedly reduced both AMH and E_2_ secretion compared with the control group (*p* < 0.001), indicating impairment of GC endocrine function. Co-treatment with hMG partially increased hormone levels relative to CTX alone; however, the extent of recovery remained limited. Treatment with naïve AMSC-sEVs (10^8^ particles/mL) resulted in modest increases in AMH and E_2_ levels under CTX-treated conditions, but the magnitude of recovery was relatively small.

In contrast, primed AMSC-sEVs significantly increased the secretion of both AMH and E_2_. Primed AMSC-sEVs at 10^4^ particles/mL elevated AMH levels compared with the CTX group (*p* < 0.001), while treatment at 10^8^ particles/mL further increased AMH secretion, reaching the highest levels among all groups (*p* < 0.001). A similar pattern was observed for E_2_, where primed AMSC-sEVs significantly increased E_2_ levels at both 10^4^ and 10^8^ particles/mL (*p* < 0.001 vs. CTX), with greater effects observed at the higher dose.

Co-treatment with primed AMSC-sEVs and hMG also increased AMH and E_2_ levels compared with CTX + hMG treatment alone (*p* < 0.001). However, the magnitude of increase was comparable to that observed with primed AMSC-sEVs alone, indicating that hMG did not provide additional enhancement of hormone secretion in the presence of primed AMSC-sEVs.

Collectively, these results demonstrate that primed AMSC-sEVs restore endocrine function in CTX-treated granulosa cells, as evidenced by increased AMH and E_2_ secretion, whereas naïve AMSC-sEVs and hMG alone show relatively limited effects.

## 3. Discussion

Gonadotropin stimulation with hMG remains a clinically relevant approach in assisted reproductive practice because it provides exogenous FSH/LH activity to promote follicular growth and steroidogenesis [26,27]. However, its clinical utility is inherently dependent on the presence of functionally competent granulosa cells that retain sufficient receptor expression, mitochondrial integrity, and steroidogenic capacity [28,29]. In the setting of chemotherapy-induced ovarian injury, this prerequisite becomes a major limitation. The present model should be interpreted as an acquired, CTX-induced POI-like granulosa cell dysfunction model. It does not reproduce the full clinical heterogeneity of POI, particularly genetically mediated forms. Once granulosa cells have undergone substantial mitochondrial impairment and downregulation of gonadotropin-related receptors, receptor-driven stimulation alone is unlikely to fully restore endocrine competence [30,31]. This concept is reflected in our CTX-injured model, in which hMG produced only partial improvement in AMH expression and estradiol secretion, while its effect on cell viability and mitochondrial membrane potential remained limited. These findings are consistent with clinical observations that patients with diminished ovarian reserve, poor ovarian response, or chemotherapy-related ovarian damage often respond suboptimally to gonadotropin stimulation despite escalating doses [32,33,34].

In this study, hMG was used to represent a clinically relevant gonadotropin stimulation context rather than as the sole therapeutic driver. This distinction is important because gonadotropin-based ovarian stimulation depends on the residual functional state of granulosa cells. Accordingly, the limited effect of hMG in CTX-injured cells suggests that receptor-driven stimulation alone may be insufficient once granulosa cell viability, mitochondrial membrane potential, and AMH/FSHR-related competence have been compromised.

Within this context, the significance of primed AMSC-sEVs lies not merely in augmenting hormonal stimulation, but in addressing a more fundamental biological defect—granulosa cell injury itself. In the present study, primed AMSC-sEVs more effectively restored cell viability, mitochondrial membrane potential, and the expression of AMH and FSHR than naïve vesicles, indicating that priming enhanced the reparative bioactivity of the vesicle cargo. This distinction is mechanistically important. Whereas hMG primarily acts through receptor-mediated endocrine signaling [27,35], primed AMSC-sEVs appear to act upstream by improving the intracellular milieu required for gonadotropin responsiveness, including mitochondrial homeostasis and preservation of granulosa cell functional identity [36,37,38]. These findings are consistent with accumulating evidence demonstrating that MSC-derived sEVs exert therapeutic effects through miRNA-mediated regulation of apoptosis, mitochondrial function, and steroidogenesis [36,37,38]. Thus, the principal value of primed AMSC-sEVs may not be to generate a simple additive effect with hMG under all conditions, but rather to restore the cellular competence upon which hormonal stimulation depends.

Importantly, our data do not support a clear synergistic interaction between primed AMSC-sEVs and hMG. Although combined treatment remained superior to CTX or CTX + hMG alone in several readouts, the addition of hMG did not consistently enhance the protective effect of primed AMSC-sEVs beyond that achieved by primed AMSC-sEVs alone. This was particularly evident in the mitochondrial membrane potential assay, in which the 10^8^ primed AMSC-sEV group showed no further improvement after hMG co-treatment and even exhibited a reduced response compared with primed AMSC-sEVs alone. Accordingly, the biological interpretation should be cautious: hMG may provide limited supportive endocrine stimulation, but it does not appear to be the dominant driver of recovery once substantial cellular injury has been established. Rather, primed AMSC-sEVs constitute the main restorative component in this model. This refinement is important because it more accurately defines the therapeutic hierarchy between endocrine stimulation and regenerative vesicle-based repair.

From a translational perspective, these findings suggest a clinically meaningful repositioning of hMG. In current practice, hMG is often used to recruit follicles and support steroidogenesis, but its efficacy is constrained when the ovarian microenvironment is already compromised [32,33,39]. Our results raise the possibility that primed AMSC-sEVs could serve as a biological conditioning strategy to improve the functional state of injured granulosa cells before or alongside gonadotropin exposure. In this framework, primed AMSC-sEVs would not simply “boost” hMG action in a pharmacologic sense; instead, they may help re-establish the cellular and mitochondrial prerequisites needed for a more effective endocrine response. Such a concept is particularly relevant for women with chemotherapy-induced ovarian insufficiency or poor ovarian responsiveness, in whom conventional gonadotropin stimulation alone is frequently inadequate [30,31,32,34,40].

The broader significance of this study therefore lies in its shift from a purely stimulatory paradigm to a restorative one. hMG represents a conventional endocrine stimulus, but its action is limited by the biological condition of the target cell. Primed AMSC-sEVs, by contrast, target the injured cellular state itself. The ability of primed AMSC-sEVs to improve mitochondrial function, preserve granulosa cell-associated markers, and restore hormone secretion indicates that they may fill an important therapeutic gap that conventional gonadotropin-based approaches cannot adequately address [36,37,38]. Future studies should further define the optimal timing and clinical context for incorporating primed AMSC-sEVs into gonadotropin-based ovarian stimulation, including their potential use before, during, or after gonadotropin exposure to support granulosa cell recovery and improve ovarian functional outcomes in POI-relevant settings.

Several limitations should be noted. First, the present model represents an acquired, CTX-induced POI-like granulosa cell dysfunction state and does not recapitulate the full etiologic spectrum of human POI, particularly genetically mediated forms. Therefore, validation in additional POI-relevant models, including genetic, autoimmune, idiopathic, or non-iatrogenic models, will be needed to define the broader applicability of primed AMSC-sEVs. Second, although all treatments were performed under 0.25% charcoal-stripped FBS conditions using the same serum lot, EV-depleted FBS was not used in this study. Residual serum-derived vesicles may therefore represent a potential background factor. Future studies using EV-depleted FBS or chemically defined serum-free conditions will be required to further validate the vesicle-specific effects. Despite these limitations, the present findings support primed AMSC-sEVs as the principal restorative component in this CTX-induced POI-like granulosa cell model and provide a basis for further evaluation in more physiologically and etiologically diverse POI systems.

## 4. Materials and Methods

### 4.1. Reagents and Cell Lines

Purchased human granulosa cells (HGL5/hGCs; Applied Biological Materials Inc., Richmond, BC, Canada) were used in this study. The HGL5 cell line is an immortalized granulosa cell model established by transfecting primary human GCs with Simian Virus 40 (SV40) large T antigen, which permits sustained proliferation while preserving core GC phenotypes. HGL5 cells express key ovarian markers, including follicle-stimulating hormone receptor (FSHR), luteinizing hormone/choriogonadotropin receptor (LHCGR), and anti-Müllerian hormone (AMH), and are widely employed in reproductive biology studies of steroidogenesis, follicular signaling, and gonadotropin responsiveness. Cells were cultured in DMEM/F-12 (Thermo Fisher Scientific Inc., Waltham, MA, USA) supplemented with 10% FBS and 1% penicillin–streptomycin (Corning, Glendale, AZ, USA) at 37 °C in a humidified atmosphere containing 5% CO_2_. Before treatment, cells were washed once with PBS and incubated for 16 h in medium containing 0.25% charcoal-stripped FBS (Cat. No. A3382101, Thermo Fisher Scientific Inc.). Naïve and primed avian mesenchymal stem cell-derived small extracellular vesicles (naïve AMSC-sEVs and primed AMSC-sEVs) were supplied by Ascension Medical Biotechnology CO., LTD. (Taipei City, Taiwan) and prepared as previously described [25]. Primed AMSC-sEVs were generated from AMSCs after exposure to *Polygonum multiflorum* Thunb. extract under serum-free, chemically defined conditions. Briefly, AMSCs were treated with 10 μg/mL extract of *P. multiflorum* Thunb. for 48 h at 37 °C in a humidified incubator containing 5% CO_2_. Conditioned medium was then collected and processed by sequential centrifugation, filtration, tangential flow filtration, and ultrafiltration as described. Cyclophosphamide (CTX; Merck, Louis, MO, USA) was used to induce POI-like granulosa cell dysfunction. Human menopausal gonadotropin (hMG) was purchased from IBSA (Meriofert^®^, 75 IU; Institut Biochimique S.A., Lamone, Switzerland). For hormone assays, estradiol (E_2_; Item No. 501890) was measured using a commercial ELISA kit (Cayman Chemical, Ann Arbor, MI, USA), and AMH was quantified using the Human AMH ELISA Kit (ab267629; Abcam, Cambridge, UK). The use of HGL5/hGCs was approved by the Institutional Review Board of the Tri-Service General Hospital and National Defense Medical University (TSGHIRB No. C202405116).

### 4.2. Establishment of the CTX-Induced POI-like Granulosa Cell Dysfunction Model

To establish an in vitro POI-like granulosa cell dysfunction model, hGCs were exposed to CTX at a final concentration of 2 μM for 48 h in medium containing 0.25% charcoal-stripped FBS. This concentration and treatment duration were selected based on our previous study [25], in which 2 μM CTX reproducibly reduced ovarian function-associated gene expression in hGCs while preserving enough cell viability for subsequent restoration assays. After the initial 48 h CTX exposure, the medium was replaced with fresh experimental medium, and cells were subjected to the indicated secondary treatments for an additional 48 h. For groups involving sustained injury, CTX was reintroduced at the same concentration during the second treatment phase.

### 4.3. Experimental Design and Treatment Groups

For rescue experiments, hGCs were assigned to the following treatment conditions: untreated control, CTX alone, hMG alone, CTX plus hMG, CTX plus naïve AMSC-sEVs, CTX plus low-dose primed AMSC-sEVs, CTX plus high-dose primed AMSC-sEVs, CTX plus hMG plus naïve AMSC-sEVs, CTX plus hMG plus low-dose primed AMSC-sEVs, and CTX plus hMG plus high-dose primed AMSC-sEVs. The CTX concentration of 2 μM was selected based on our previous study and preliminary dose–response results showing reproducible suppression of granulosa cell functional markers while maintaining sufficient viability for subsequent rescue assays. Naïve AMSC-sEVs were administered at 1 × 10^8^ particles/mL as the reference vesicle dose. Primed AMSC-sEVs were applied at either 1 × 10^4^ particles/mL or 1 × 10^8^ particles/mL to evaluate whether priming enhanced vesicle potency and allowed functional recovery at a lower particle exposure. Human menopausal gonadotropin was used at a final concentration of 1 IU/mL during the second 48 h treatment phase, based on dose–response testing showing the most consistent partial restoration of AMH and FSHR expression in CTX-treated hGCs. Equivalent volumes of PBS and/or DMSO were added to the corresponding control groups where appropriate. This treatment design was based on the previously established two-phase CTX injury/recovery model and was adapted in the present study to examine hMG-associated functional recovery in combination with primed AMSC-sEVs. Each treatment condition was performed with six technical replicates, and all experiments were independently repeated three times.

### 4.4. Cell Viability Assay

Cell viability was assessed using the Cell Counting Kit-8 (CCK-8; DOJINDO Laboratories, Kumamoto, Japan). Cells were seeded into 96-well plates at a density of 5 × 10^3^ cells/well in 100 μL of complete medium containing 10% FBS and allowed to attach for 24 h. The medium was then replaced with DMEM/F-12 containing 0.25% charcoal-stripped fetal bovine serum, and the cells were subjected to this for 16 h before treatment according to the experimental design. At the end of treatment, culture medium was replaced with fresh medium containing 10% CCK-8 reagent, and the cells were incubated for 2 h at 37 °C. Absorbance was measured at 450 nm using a VersaMax microplate reader (Molecular Devices, San Jose, CA, USA), and cell viability was expressed relative to the untreated control group.

### 4.5. Quantitative Real-Time PCR

Total RNA was extracted from treated hGCs using the Quick-RNA Miniprep Kit (Zymo Research Corporation, Irvine, CA, USA) with on-column DNase I digestion. Reverse transcription was performed using 0.5 μg of total RNA and the PrimeScript™ RT Reagent Kit (Takara Bio Inc., Shiga, Japan). Quantitative real-time PCR was carried out using the QuantiFast SYBR Green PCR Kit (Qiagen, Hilden, Germany) on a Rotor-Gene Q Real-Time PCR Detection System (Qiagen). Thermal cycling consisted of an initial denaturation at 95 °C for 5 min, followed by 45 cycles at 95 °C for 10 s and 60 °C for 30 s. Relative expression levels of AMH, FSHR, and LHCGR were analyzed as principal functional markers of granulosa cells. Gene expression was normalized to 18S rRNA, and relative changes were calculated using the 2^−ΔΔCt^ method. Primer sequences are listed in Table 1.

### 4.6. Mitochondrial Membrane Potential Assay

Mitochondrial membrane potential (ΔΨm) was assessed using tetramethylrhodamine, methyl ester (TMRM). The staining reagent used in this study was Image-iT™ TMRM Reagent (Thermo Fisher Scientific, Cat. No. I34361), which was supplied as a 100 μM stock solution in DMSO. According to the manufacturer’s instructions, the stock solution was freshly diluted in culture medium to a final working concentration of 100 nM. After completion of the indicated treatments, hGCs were incubated with TMRM for 30 min at 37 °C under light-protected conditions. Cells were then gently washed with PBS, and fluorescence intensity was measured immediately using a CLARIOstar^®^ Plus microplate reader (BMG LABTECH, Ortenberg, Germany) at an excitation wavelength of 548 nm and an emission wavelength of 574 nm. Fluorescence values were normalized to the untreated control group and expressed as relative mitochondrial membrane potential. Reduced TMRM fluorescence intensity was interpreted as mitochondrial membrane depolarization, whereas restoration of fluorescence signal after treatment was regarded as recovery of mitochondrial membrane potential. This assay was designed as a quantitative plate-reader-based measurement rather than an imaging-based analysis.

### 4.7. Statistical Analysis

Data are presented as mean ± standard deviation (SD). Each experiment included six technical replicates per group and was independently repeated three times. Statistical analyses were performed using GraphPad Prism version 9.0.0 (121) for Windows (GraphPad Software, San Diego, CA, USA) or IBM SPSS Statistics version 19.0 (IBM Corp., Armonk, NY, USA), as appropriate. Comparisons among multiple groups were analyzed by one-way analysis of variance (ANOVA) followed by an appropriate post hoc test. A *p* value of less than 0.05 was considered statistically significant.

## 5. Conclusions

Primed AMSC-sEVs effectively attenuated CTX-induced granulosa cell injury by restoring cell viability, mitochondrial membrane potential, granulosa cell-associated gene expression, and hormone secretion. Compared with hMG, primed AMSC-sEVs showed a more pronounced restorative effect under cytotoxic conditions, whereas co-treatment with hMG did not provide consistent additional benefit. These findings suggest that primed AMSC-sEVs act primarily by improving granulosa cell homeostasis rather than by simply enhancing gonadotropin signaling. Collectively, primed AMSC-sEVs represent a promising cell-free strategy for ovarian functional restoration in chemotherapy-related ovarian injury and other conditions associated with impaired granulosa cell competence.

## Figures and Tables

**Table 1:** Primer sequences for *Homo sapiens*.

Gene Name	Forward	Backward	Accession No.
Human *AMH*	GCCTTGCCCTCTCTACGGC	TGTTGGCTCCCAGGTCACTTC	NM_000479.5
Human *FSHR*	GGAACCCAACTAGATGCAGTGA	CAGAGGCTCCGTGGAAAACA	M65085.1
Human 18s	GTAACCCGTTGAACCCCATT	CCATCCAATCGGTAGTAGCG	NR_003286

## Data Availability

The datasets generated and/or analyzed during the current study are available from the corresponding author upon reasonable request.

## Supplementary Materials

The following supporting information can be downloaded at: https://www.mdpi.com/article/10.3390/ijms27114934/s1.

## Abbreviations

The following abbreviations are used in this manuscript:

sEVs	small extracellular vesicle
CTX	cyclophosphamide
hMG	human menopausal gonadotropin
AMH	anti-Müllerian hormone
FSHR	follicle-stimulating hormone receptor
POI	premature ovarian insufficiency
