# Supplementary material for: Primed Avian Mesenchymal Stem Cell-Derived Small Extracellular Vesicles Restore Granulosa Cell Homeostasis in a CTX-Induced POI-like Dysfunction Model Under Human Menopausal Gonadotropin Stimulation
Source: Int J Mol Sci. 2026 May 29;27(11):4934. doi: 10.3390/ijms27114934 (PMC13257334; doi:10.3390/ijms27114934)
Supplement: Supplementary file 1 [file ijms-27-04934-s001.zip › ijms-4296194-Table S1.pdf]

**Group**	**Non sEVs**			**Naïve sEVs**		**Primed sEVs**			
**AMSC sEVs (particles/ml)**	—	—	—	10^8	10^8	10^4	10^4	10^8	10^8
**CTX (2 μM)**	—	+	+	—	+	—	+	—	+
**hMG (1 IU/ml)**	—	—	+	—	+	—	+	—	+
**AMH (ng/ml)**	0.0636±0.0024	0.0103±0.0009	0.0765±0.0029	0.0633±0.0020	0.0180±0.0030	0.0885±0.0061	0.0918±0.0044	0.1255±0.0077	0.2078±0.0202
**E_2 (pg/ml)**	2.124±0.059	1.119±0.029	2.926±0.245	2.145±0.119	3.909±0.363	3.028±0.229	7.008±0.439	8.162±0.298	7.075±0.266

Actual AMH and E_2 concentrations are presented as mean ± SD.
